# Receptor-Side Neuroimmune Remodeling in IBD: Participant-Level Associations with Metabolite-Sensing Programs and Colorectal Neoplasia Context

**DOI:** 10.3390/ijms27209179

**Published:** 2026-10-16

**Authors:** Hui Hu, Kemin Xu, Xu Yuan, Zhang Lei, Yanjun Lu

**Affiliations:** 1 Department of Laboratory Medicine, Tongji Hospital, Tongji Medical College, Huazhong University of Science and Technology, Wuhan 430030, China; 2 Department of Infectious Diseases, Tongji Hospital, Tongji Medical College and State Key Laboratory for Diagnosis and Treatment of Severe Zoonotic Infectious Disease, Huazhong University of Science and Technology, Wuhan 430030, China; 3 Department of Oncology, The Central Hospital of Wuhan, Tongji Medical College, Huazhong University of Science and Technology, Wuhan 430014, China; 4 Key Laboratory for Molecular Diagnosis of Hubei, Wuhan Central Hospital, Tongji Medical College, Huazhong University of Science and Technology, 16 Gusaoshu Road, Wuhan 430812, China

**Keywords:** inflammatory bowel disease, gut microbiota, metabolite sensing, receptor-side neuroimmune remodeling, mucosal compartments, colorectal neoplasia

## Abstract

Background: Microbial metabolite sensing and neuroimmune signaling are increasingly recognized as interacting components of the mucosal response in inflammatory bowel disease (IBD). However, their organization across individuals and the cellular compartments carrying the relevant receptor machinery remain incompletely understood. We investigated whether metabolite-sensing host programs covary with neuroimmune receptor-side mucosal states at the participant level and whether the corresponding receptor transcripts map to defined mucosal compartments. Methods: HMP2 mucosal transcriptomes were analyzed at the participant level to evaluate prespecified relationships between metabolite-sensing and neuroimmune receptor-side host programs. Receptor-side neuroimmune remodeling was operationalized as cross-sectional participant-level host-program organization together with compartmental localization of the corresponding receptor transcripts, rather than longitudinal change or receptor activation. The stability and interpretation of these relationships were examined through diagnosis/inflammation adjustment, participant-grouped explanatory modeling, and tissue-composition sensitivity analyses. Complementary metabolomic, single-cell, UC, and colorectal-neoplasia datasets were used to examine synchronized metabolic associations, receptor-transcript localization, and the broader disease context of the identified mucosal programs. The microbiome, metabolomic, and transcriptomic datasets were obtained from multiple independent public cohorts. Results: Among 251 HMP2 biopsies from 90 participants, 13 of 20 prespecified host–program associations passed FDR correction in participant-mean analyses, and 13 of 20 remained supported after diagnosis/inflammation adjustment, with 12 associations shared between the two analyses. The strongest relationships connected the SCFA receptor/barrier-response program with the inflammation-associated reactive/glial-adjacent state (ρ = 0.727) and the CGRP/adrenomedullin-family receptor-side score (ρ = 0.552), and connected the AHR-response context with the same receptor-side score (ρ = 0.531). Single-cell analyses localized CALCRL, RAMP2, and RAMP3 mainly to endothelial cells, VIPR1 to epithelial cells, and VIPR2 to stromal cells. The attenuation of incremental model fit after composition-proxy conditioning further indicated that this organization is closely embedded in mucosal tissue ecology. Matched metabolomic and premalignant-cohort analyses provided complementary evidence on the scope of this framework but did not extend it to synchronized metabolite coupling or neoplastic progression. Conclusions: Participant-level associations and compartment-resolved receptor localization support a cross-sectional receptor-side neuroimmune remodeling framework in IBD. The convergence of host-program organization and cellular localization prioritizes specific receptor–compartment relationships for spatial validation, functional receptor perturbation, and prospectively matched stool–mucosal studies.

## 1. Introduction

Inflammatory bowel disease (IBD) establishes a chronically injured mucosal ecosystem in which microbial dysbiosis, metabolic disturbance, epithelial barrier dysfunction, and immune activation progressively reinforce one another [1]. A Global Burden of Disease 2021 analysis estimated 375,140 incident IBD cases (95% uncertainty interval [UI], 327,686–436,925) and 1,510,784 IBD disability-adjusted life-years (1,308,508–1,750,363) worldwide in 2021, with an age-standardized DALY rate of 18.07 per 100,000 [2]. Large-scale multi-omic studies have demonstrated that IBD is not defined by a single microbial taxon or isolated inflammatory pathway, but rather by coordinated alterations across microbial composition, functional potential, metabolite profiles, host transcriptional states, and clinical activity [3]. HMP2/IBDMDB established this principle by profiling the gut microbial ecosystem across multiple molecular layers in IBD [4], and cross-cohort microbiome–metabolome integration has further confirmed that IBD-associated metabolic signatures are reproducible yet heterogeneous across populations [5]. More recent work has extended this view: strain-resolved metagenomics indicates that IBD-associated bacteria can represent disease-adapted lineages rather than transient species-level markers [6], and large transethnic microbiome studies demonstrate that fecal bacterial panels can classify IBD across diverse populations [7]. While microbiome profiling in IBD has advanced greatly, how luminal dysbiosis is organized with mucosal injury and multicompartment host responses remains poorly defined.

The intestinal barrier constitutes the anatomical and functional interface through which luminal signals are filtered into immune and stromal responses. Recent barrier-focused work frames the epithelium, mucus layer, antimicrobial peptides, and lamina propria immune system as a disease-defining boundary whose disruption contributes to IBD, colorectal cancer, and systemic inflammatory conditions [8]. Barrier integrity and immune activation are also shaped by neural signals, positioning the gut–brain–immune axis at the center of current IBD research. Recent syntheses indicate that microbial products and metabolites can shape immune-cell phenotypes and neuronal sensitivity, whereas sensory and enteric neuronal mediators reciprocally regulate local immune homeostasis [9]. In bulk mucosal biopsies and tumor single-cell atlases, canonical enteric neurons are sparsely captured or absent; a neuroimmune signal in these data is therefore more plausibly interpreted as receptor-side or downstream host-response activity in epithelial, stromal, myeloid, or endothelial-associated compartments. Human and mouse enteric nervous system atlases have mapped specialized neuronal and glial diversity [10], and investigations of enteric glial biology support its relevance to barrier function [11]. Accordingly, we interpret the CXCL10/NOS2/S100A10 program as an inflammation-associated reactive/glial-adjacent response module, in which “glial-adjacent” denotes biological context rather than enteric-glial identity or evidence of altered glial abundance or activation.

IBD-associated colorectal cancer develops in the context of chronic inflammation, epithelial injury, dysplasia surveillance, stromal remodeling, and altered immune organization. Contemporary reviews emphasize both the clinical burden and the mechanistic complexity of inflammation-driven neoplasia [12]. Cancer neuroscience adds another dimension by demonstrating that neurotransmitter and neuropeptide signaling can shape colorectal tumor biology and immune responses [13], and intestinal immunology studies indicate that neuropeptides such as α-CGRP can modulate immune-cell responses [14]. These observations suggest that CAC-related neuroimmune biology is most appropriately considered within a broader inflammatory, epithelial, stromal, myeloid, and vascular tissue ecology. Sporadic tumors, cross-sectional colitis biopsies, receptor-localization datasets, and CAC tissues nevertheless represent complementary contexts rather than successive stages of a temporal model.

The key gap is not whether dysbiosis, metabolites, or neuroimmune mediators are altered in IBD, but which mucosal compartments receive these signals and how the corresponding receptor-side programs are organized in chronically injured tissue. We therefore built an integrative multi-omic analysis beginning with IBD-associated microbial and metabolic context, moving to participant-level mucosal host responses and single-cell localization, and then examining UC, sporadic CRC, published CAC spatial, and exploratory AOM-DSS contexts. Our hypothesis was that IBD-associated microbial–metabolic disturbance is accompanied by a receptor-side neuroimmune remodeling pattern that can be characterized through participant-level host-program associations, localized to defined tissue compartments, and tested in future matched spatial and functional studies. Here, receptor-side neuroimmune remodeling refers to the cross-sectional organization of receptor-associated mucosal programs together with the compartmental localization of relevant receptor transcripts, rather than longitudinal change. The contributing datasets were analyzed as complementary evidence layers, not as successive stages of one patient-level disease course.

## 2. Results

**Figure 1:**
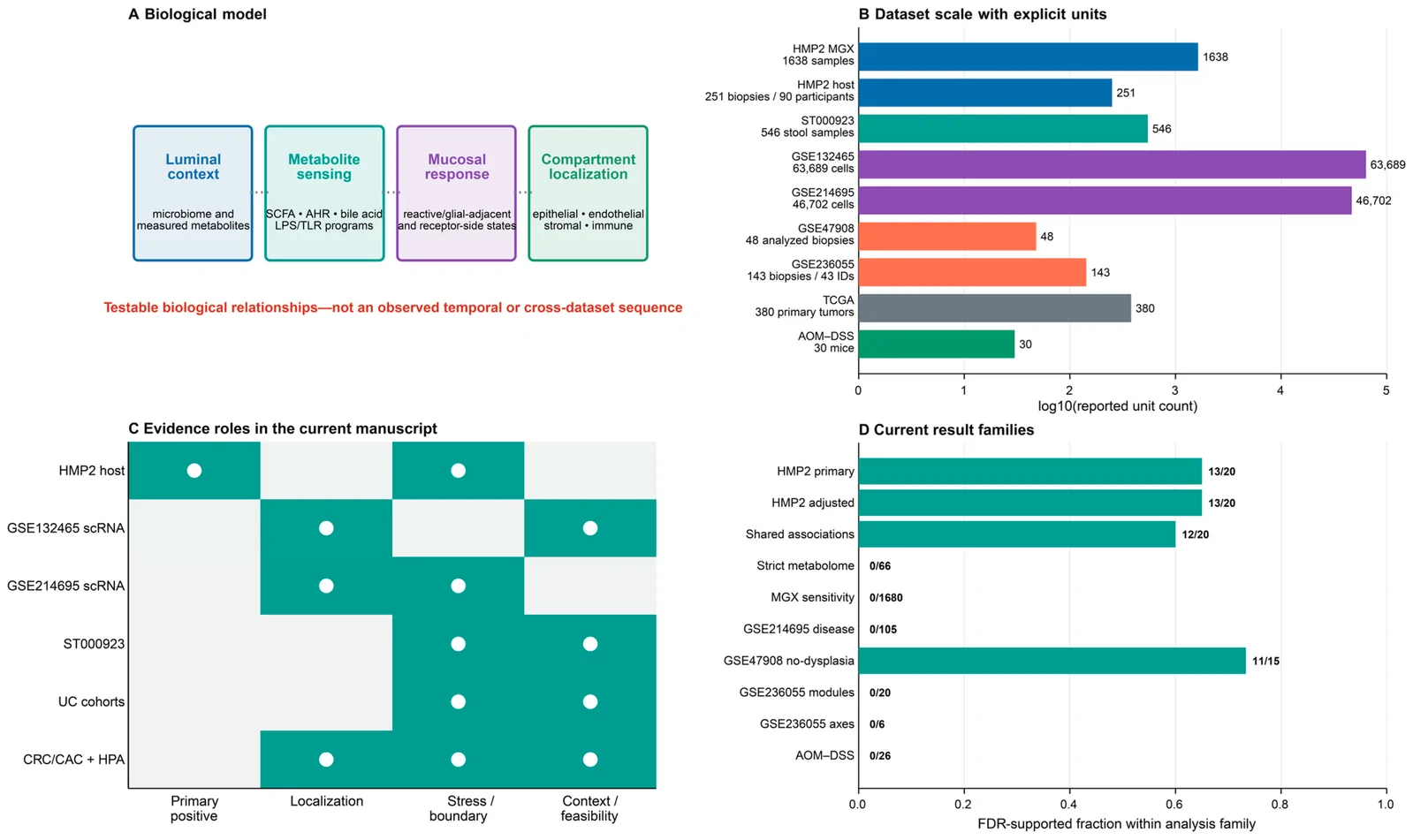
Biological model and analytical design for a receptor-side mucosal response hypothesis. (**A**) Four testable relationships connect luminal context, metabolite-sensing host programs, receptor-side/reactive mucosal states, and receptor-bearing compartments; Gray dotted connectors indicate hypothesized relationships between the conceptual components, rather than observed temporal progression, causal links, or cross-dataset transitions. (**B**) Dataset scales are shown with explicit independent units, including 380 unique primary TCGA tumors. (**C**) The evidence-role matrix separates primary host covariance, single-cell localization, sensitivity analyses, and contextual or feasibility layers; HMP2 bulk host expression is not treated as compartment-localization evidence. (**D**) FDR-supported counts are summarized for the prespecified analysis families, including 13/20 primary, 13/20 adjusted, 12/20 shared, 0/66 strict metabolome, 0/1680 MGX, 0/105 GSE214695 disease-comparison, 11/15 dysplasia-excluded GSE47908, 0/20 and 0/6 GSE236055, and 0/26 AOM–DSS results.

Figure 1 outlines the study design and the distinct contribution of each dataset. We first describe the luminal microbial and measured-metabolite context, then present the participant-level host associations and receptor-transcript localization, followed by analyses of UC and colorectal–neoplasia tissue context. Table 1 summarizes the datasets and analysis units, Table 2 reports the principal quantitative results, and Table 3 distinguishes the interpretations supported by each analysis from questions that remain untested.

### 2.1. Microbial and Metabolic Layers Define the Luminal Context in IBD

**Figure 2:**
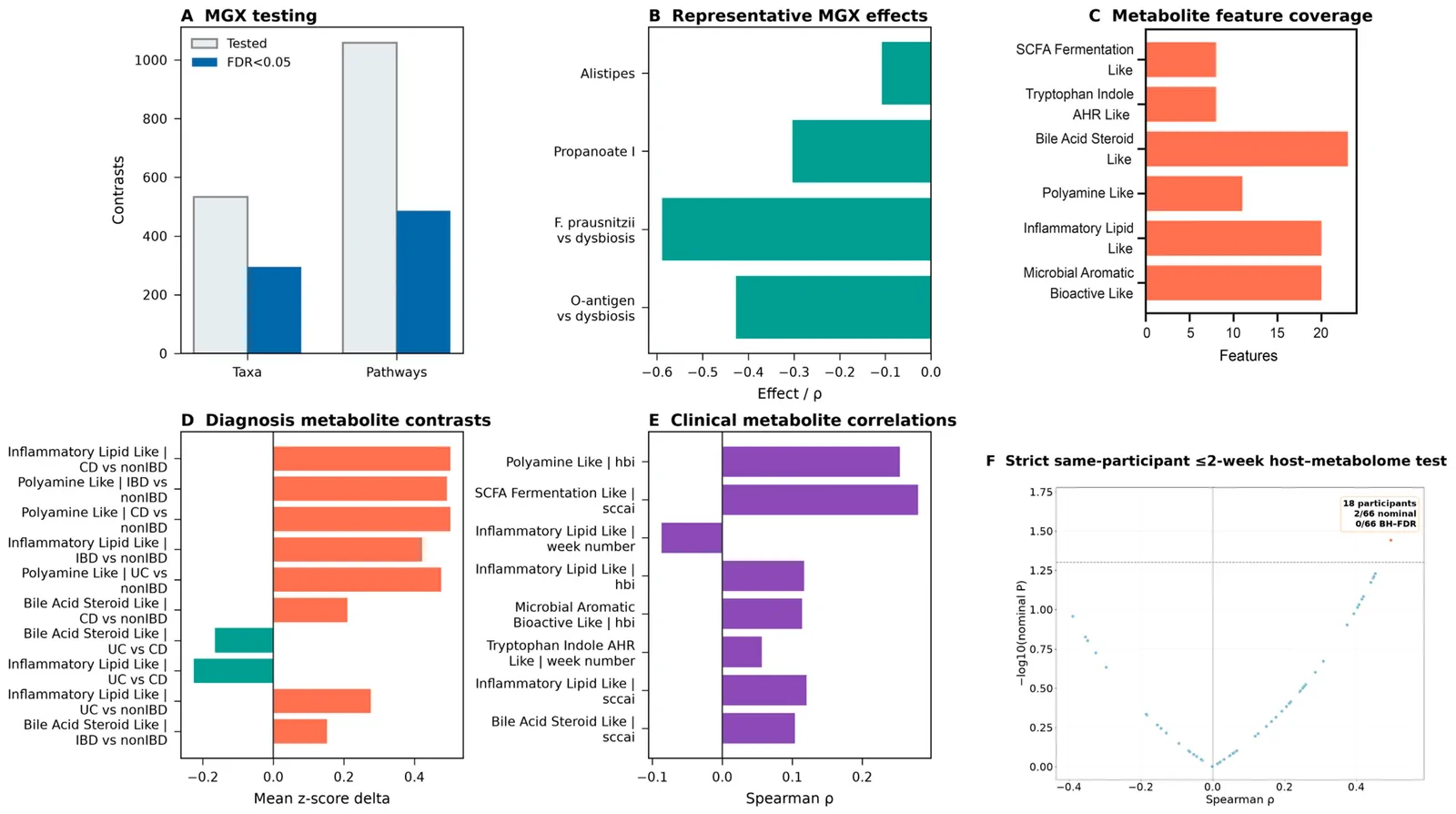
IBD-associated luminal microbial and measured fecal metabolite context. (**A**) Tested and FDR-supported HMP2 metagenomic diagnosis contrasts. (**B**) Representative taxonomic and pathway-potential effects. (**C**) Coverage of the post hoc name-regex metabolite categories. (**D**) Diagnosis-associated category contrasts. (**E**) Descriptive clinical correlations. (**F**) Strict same-participant ≤2-week host–metabolome analysis (18 participants; 2/66 nominal, 0/66 BH-FDR). Each dot represents one of 66 Spearman correlation tests between six fecal metabolite category scores and 11 mucosal host-module scores. The horizontal dashed line indicates the nominal *p* = 0.05 threshold (−log10 *p* ≈ 1.30), whereas the vertical solid line marks ρ = 0. Metagenomic functional potential and measured fecal metabolites are distinct luminal layers; fecal abundance is not mucosal exposure, and neither layer establishes host mediation.

We first characterized the luminal environment in which intestinal-wall responses arise. HMP2 metagenomics encompassed 1638 samples and revealed broad diagnosis-associated differences in microbial taxonomic composition and functional potential (Figure 2A,B; Table 1). Representative signals included lower Faecalibacterium prausnitzii and O-antigen pathway abundance with dysbiosis and lower propanoate I pathway abundance in IBD. These metagenomic findings define microbial ecological context rather than measured metabolite abundance or synchronized mucosal exposure.

Measured fecal metabolomics from ST000923 provided an orthogonal metabolic-output layer distinct from metagenomic pathway potential (Figure 2C–F). Six post hoc exploratory category scores summarized 87 of 596 measured features across 546 stool samples. Under the strict same-participant ≤2-week criterion, 18 participants contributed paired stool and mucosal observations; no category-to-host-module association passed BH-FDR correction under either tie-handling approach (0/66 in both analyses), although 2 nominal associations occurred with the source-row-order rule and 6 after participant-week median aggregation (Table 2; Supplementary Table S3). Alternative aggregation, curated-exclusion, leave-one-feature-out, and feature-level analyses did not alter the multiplicity-adjusted conclusion. Thus, the data show cohort-level microbial and metabolic variation, whereas the strict temporally matched subset did not establish synchronized fecal-metabolite–mucosal-host covariation.

### 2.2. Participant-Level Associations Characterize Receptor-Side Neuroimmune Remodeling in the IBD Mucosa

**Figure 3:**
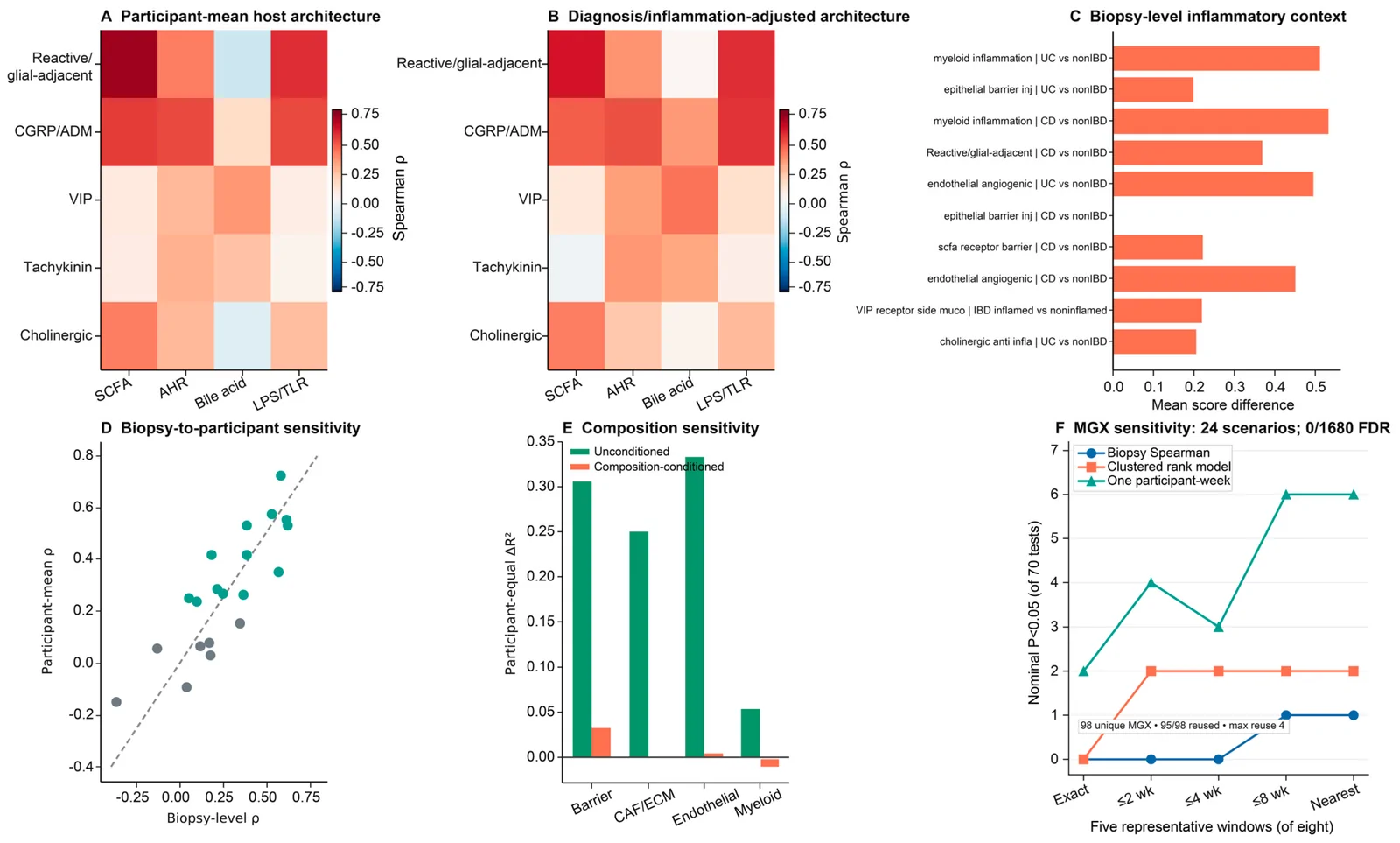
Participant-aware HMP2 host organization and composition sensitivity. (**A**) Participant-mean correlations between four metabolite-sensing programs and five receptor-side or reactive mucosal programs; 13/20 passed BH-FDR. (**B**) Diagnosis/inflammation-adjusted participant-level correlations; 13/20 passed BH-FDR, with 12 shared with the primary analysis. (**C**) Biopsy-level inflammatory and injury/repair contrasts provide tissue-state context. (**D**) Biopsy-level versus participant-mean estimates. (**E**) Participant-grouped direct-versus-combined ΔR^2^ before and after composition-proxy conditioning; conditioned increments were 0.034, −0.001, 0.005, and −0.010. (**F**) Five representative windows visualize three analytical strategies; the complete eight-window × three-strategy family comprised 24 scenarios and 1680 tests, none of which passed BH-FDR. Ninety-eight unique MGX profiles were available, 95 were reused, and maximum reuse was four biopsies. This layer provides same-participant ecological context rather than synchronized exposure or mediation.

The primary host-side finding was a coordinated participant-level organization linking metabolite-sensing and neuroimmune receptor-side programs with epithelial barrier, inflammatory, reactive/glial-adjacent, stromal, and vascular states. Across 251 HMP2 biopsies from 90 participants, 13 of 20 prespecified participant-mean associations passed Benjamini–Hochberg false-discovery-rate (BH-FDR) correction; 13 of 20 remained supported after adjustment for diagnosis and inflammation status, with 12 associations shared between the two analyses (Figure 3A,B,D; Table 2; Supplementary Tables S1 and S2). The strongest relationships connected the SCFA receptor/barrier-response program with the inflammation-associated reactive/glial-adjacent module (Spearman ρ = 0.727; 95% CI, 0.602–0.817) and the CGRP/adrenomedullin-family receptor-side score (ρ = 0.552; 95% CI, 0.400–0.679). The AHR-response context was also associated with the CGRP/adrenomedullin-family score (ρ = 0.531; 95% CI, 0.364–0.681). Adjustment refined rather than uniformly strengthened the architecture: the AHR–cholinergic relationship no longer passed FDR correction, whereas the bile acid–CGRP/adrenomedullin-family relationship became supported.

Disease-state and biopsy-inflammation comparisons placed this participant-level organization within active mucosal injury: myeloid inflammation and epithelial barrier injury/repair scores were higher in UC than in non-IBD biopsies, and the inflammation-associated reactive/glial-adjacent module was higher in inflamed than in noninflamed biopsies (Figure 3C). Participant-grouped ridge analyses further examined incremental prediction among host-expression programs (Figure 3E; Table 2). Before composition conditioning, held-out participant-equal ΔR^2^ values were 0.307 for barrier injury/repair (95% participant-bootstrap CI, 0.044–1.368), 0.251 for CAF/ECM remodeling (0.109–0.510), 0.334 for endothelial angiogenesis (0.203–0.678), and 0.054 for myeloid inflammation (−0.086–0.163). After epithelial, myeloid, stromal, and endothelial expression-derived composition proxies were added to both model sets, the corresponding increments attenuated to 0.034, −0.001, 0.005, and −0.010. Thus, the positive host-expression covariance is embedded in a coordinated multicompartment tissue ecology and is not a clinical prediction model or evidence of a microbial or metabolite mechanism.

A separate timing analysis tested whether matched metagenomic profiles provided synchronized cross-layer support. Across 24 same-participant sensitivity scenarios spanning eight matching windows and three analytical strategies, none of 1680 host–MGX tests passed BH-FDR correction (Figure 3F; Supplementary Table S12). The analysis used 98 unique MGX profiles; 95 were matched to more than one biopsy, and an individual profile was reused for as many as four biopsies. The MGX layer therefore provides ecological context, not an independent temporally synchronized measure of mucosal exposure.

### 2.3. Receptor-Side Neuroimmune Programs Localize to Mucosal Recipient Compartments

**Figure 4:**
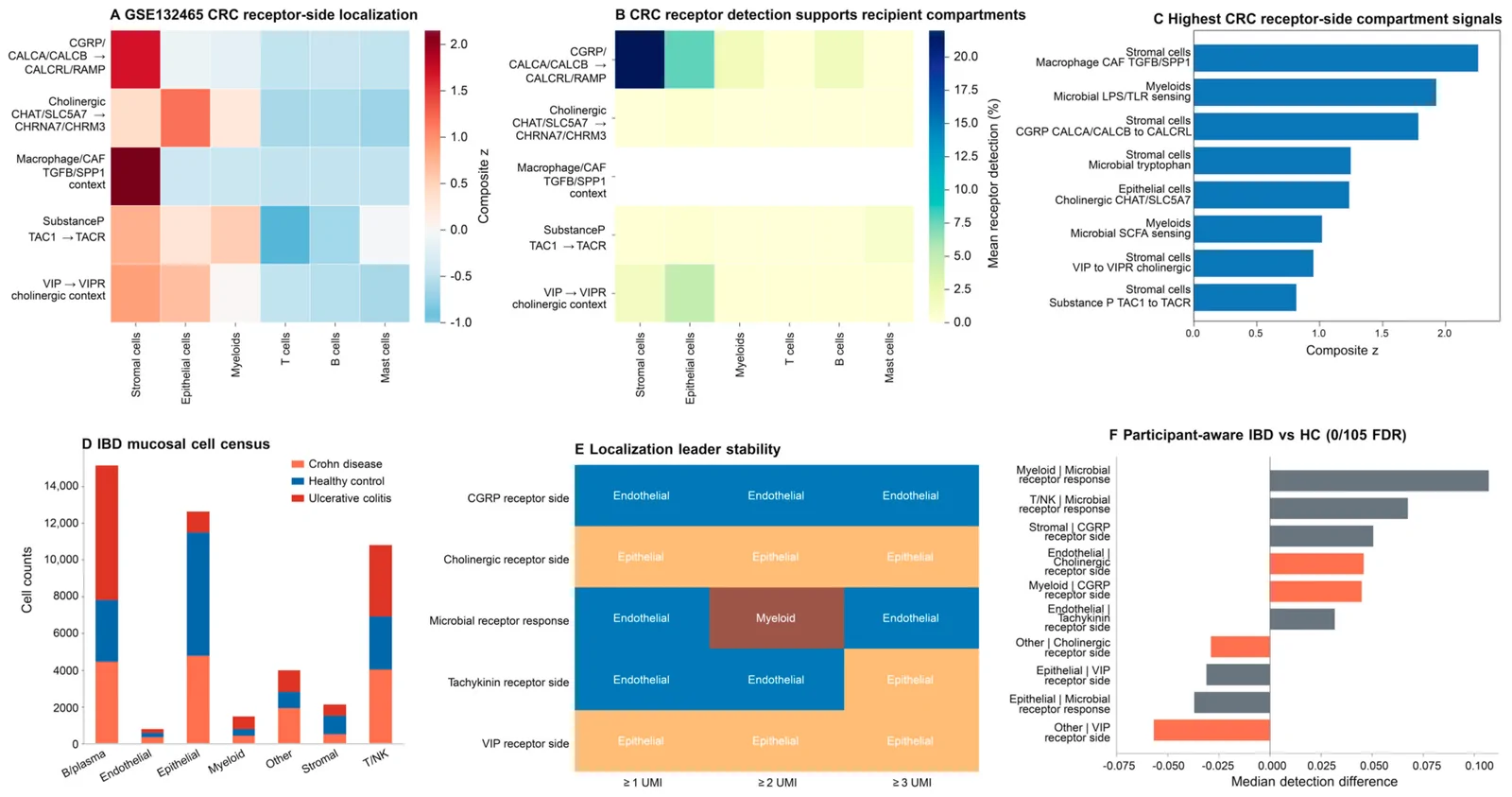
Receptor-side transcript localization in CRC and IBD mucosal compartments. (**A**–**C**) GSE132465 panels summarize receptor-side and downstream-program localization across stromal, epithelial, myeloid, T-cell, B-cell, and mast-cell compartments; this dataset does not supply the endothelial localization claim. (**D**) GSE214695 cell counts across disease groups and major mucosal compartments. (**E**) Threshold-stable localization assignments, including endothelial CGRP/adrenomedullin-family components, epithelial VIPR1, and stromal VIPR2. (**F**) Participant-aware IBD-versus-healthy detection differences; 0/105 comparisons passed BH-FDR. These panels describe transcript localization and detection, not ligand–receptor communication, signaling activity, neuronal expansion, CAC specificity, or prediction.

As a compartment-localization layer rather than a disease-origin analysis, GSE132465 single-cell summaries mapped receptor-side and downstream response programs across 63,689 CRC cells and stromal, epithelial, myeloid, T-cell, B-cell, and mast-cell compartments (Figure 4A–C; Supplementary Table S4). CGRP/adrenomedullin-family and AHR-related scores were prominent in stromal summaries, LPS/TLR-associated inflammatory expression score was most prominent in myeloid summaries, and the cholinergic receptor-side context was most evident in epithelial summaries. These patterns provide a cellular framework for the participant-level bulk-tissue covariance but do not infer physical ligand–receptor contact, receptor activation, or endothelial localization from this dataset.

GSE214695 provided a complementary receptor-transcript map in IBD mucosa, comprising 46,702 annotated cells from 18 participant-sample units (6 healthy control, 6 UC, and 6 colonic CD; Figure 4D–F; Supplementary Figure S1; Supplementary Table S8). CALCRL, RAMP2, and RAMP3 detection was highest in endothelial cells, VIPR1 localized mainly to epithelial cells, and VIPR2 to stromal cells; these leading assignments were stable across the ≥1-, ≥2-, and ≥3-UMI detection thresholds. In total, 0 of 105 participant-aware IBD-versus-healthy comparisons passed BH-FDR correction (minimum FDR, 0.106), so compartment localization was more consistent than evidence of disease-associated remodeling. Human Protein Atlas annotations identified public protein evidence and antibody resources for prioritized targets (Supplementary Figure S2; Supplementary Table S10), providing assay feasibility rather than disease-state protein validation.

### 2.4. UC Cohorts Distinguish Inflammatory-State Remodeling from Progression-Associated Change

**Figure 5:**
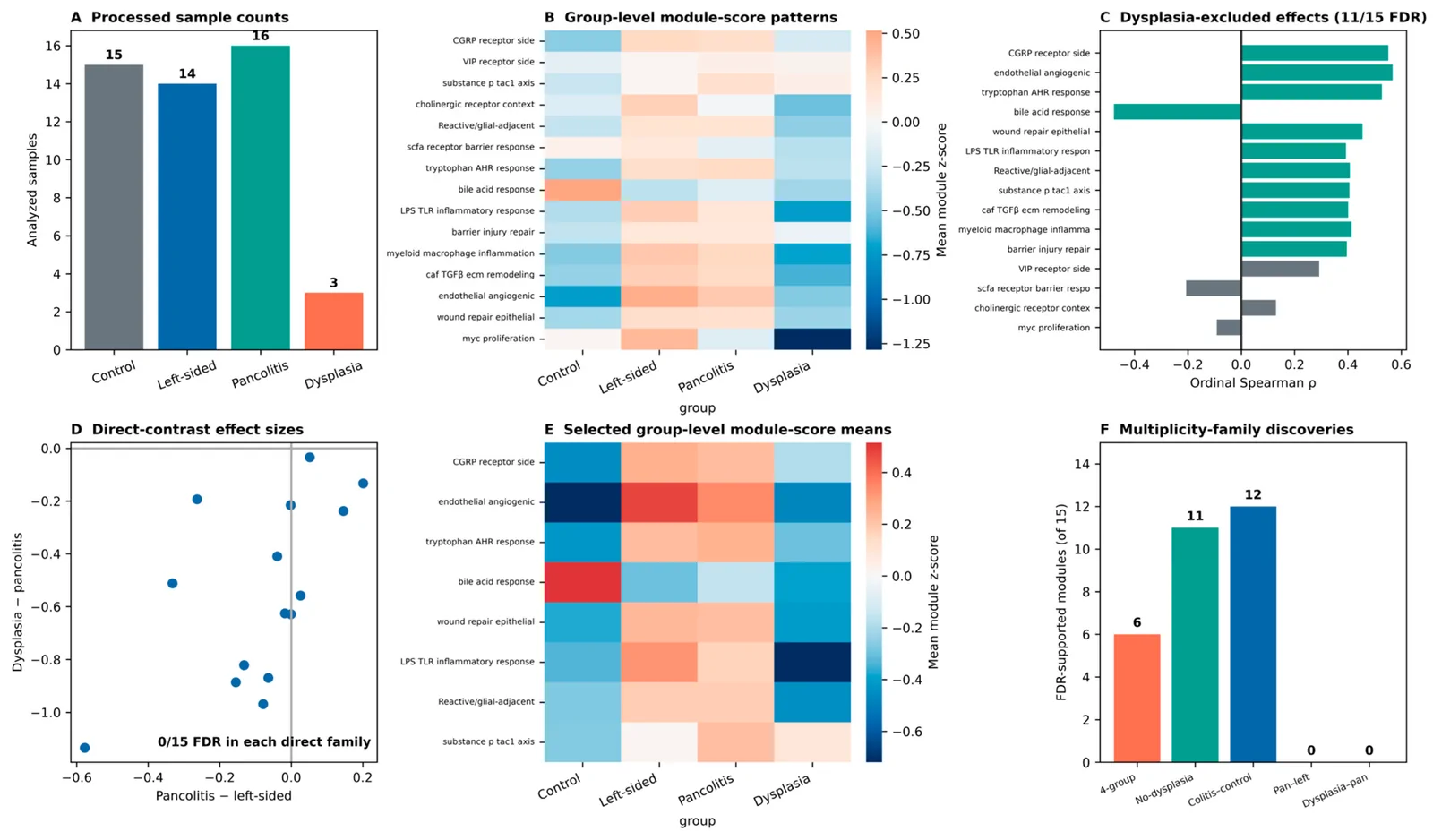
Cross-sectional UC extent and dysplasia context in GSE47908. (**A**) Processed sample counts: 15 controls, 14 left-sided colitis, 16 pancolitis, and 3 dysplasia samples. (**B**) Group-level module-score patterns. (**C**) Dysplasia-excluded ordinal effects, with 11/15 modules passing BH-FDR. Teal bars indicate FDR-significant modules (adjusted *p* < 0.05), whereas gray bars indicate non-significant modules. (**D**) Direct pancolitis-versus-left-sided and dysplasia-versus-pancolitis effect sizes; neither 15-test family yielded an FDR-supported result. (**E**) Selected group-level module-score means. (**F**) Discovery counts across five separately corrected families: 6/15 four-group ordinal, 11/15 dysplasia-excluded ordinal, 12/15 pooled colitis versus control, 0/15 pancolitis versus left-sided, and 0/15 dysplasia versus pancolitis. These cross-sectional associations do not establish trajectory, progression, prediction, or causality.

We next evaluated whether the intestinal-wall programs varied across UC tissue states. The processed GSE47908 matrix represented 48 of 60 biopsies in the complete series: 15 of 15 controls, 14 of 20 left-sided colitis samples, 16 of 19 pancolitis samples, and 3 of 6 UC-associated dysplasia samples (Figure 5A,B,E; Supplementary Figure S4; Supplementary Tables S5 and S11). After exclusion of the three dysplasia samples, 11 of 15 prespecified modules were associated with the cross-sectional control/left-sided-colitis/pancolitis ordering at BH-FDR < 0.05, with 10 positive and 1 negative effect (Figure 5C,F). In contrast, direct pancolitis-versus-left-sided-colitis testing yielded 0 of 15 FDR-supported differences, and direct dysplasia-versus-pancolitis testing also yielded 0 of 15 (Figure 5D,F). When dysplasia was retained in the four-group ordinal analysis, 6 of 15 modules passed FDR correction, but the dysplasia group contained only three processed samples. The ordinal pattern therefore primarily reflects control-versus-established-colitis separation, not a graded extent effect, a dysplasia-specific program, or longitudinal progression.

GSE236055 tested a related but distinct boundary in 143 histologically non-neoplastic biopsies from 43 deposited patient IDs, including 13 progressors and 30 nonprogressors; the series description reports 14 progressors, but a fourteenth deposited progressor ID could not be resolved. Using one median score per patient ID, 0 of 20 prespecified modules and 0 of 6 derived axes passed BH-FDR correction (Supplementary Figure S3; Supplementary Table S9). Together, the UC cohorts show cross-sectional inflammatory-state associations but no FDR-supported dysplasia-specific or participant-level progressor signal; they do not validate progression, a biomarker, or clinical prediction.

### 2.5. Colorectal Neoplasia Provides Stromal–Immune Context Rather than Cac Validation

**Figure 6:**
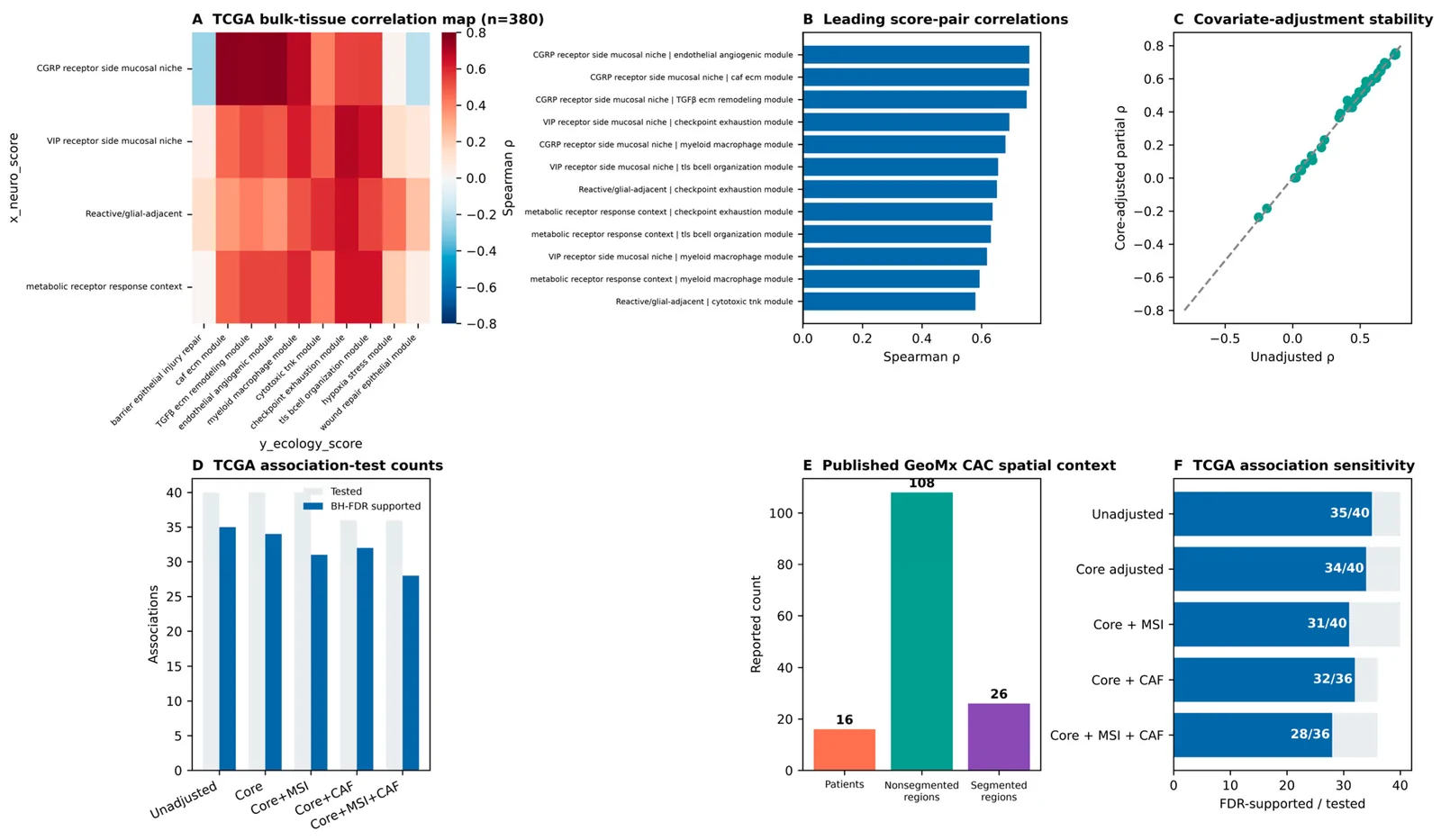
Contextual colorectal-neoplasia analyses. (**A**) TCGA COAD/READ bulk-tissue score correlations in 380 unique primary sporadic CRC tumors. (**B**) Leading score-pair correlations. (**C**) Stability of correlations after core covariate adjustment. (**D**) Tested and BH-FDR-supported association counts across sensitivity models. (**E**) Published GeoMx CAC spatial context from 16 patients, 108 nonsegmented regions, and 26 segmented regions; ROI-level matrices were unavailable for independent rescoring. (**F**) FDR-supported/tested counts across unadjusted, core-adjusted, MSI, CAF-proxy, and combined models. TCGA supplies sporadic-CRC bulk-tissue context rather than CAC validation, CMS/purity independence, causality, or tumor-cell-intrinsic inference.

After restriction to 380 unique primary COAD/READ tumors, TCGA analyses showed that receptor-side scores frequently covaried with stromal, immune, and vascular expression programs (Figure 6A–D,F; Supplementary Table S6). Of 40 tested score pairs, 35 were FDR-supported without adjustment, 34 after core covariate adjustment, and 31 in core-plus-MSI complete-case models; sensitivity models incorporating an expression-derived CAF proxy supported 32 of 36 pairs, or 28 of 36 with MSI also included. Tumor stage was available for 358 cases, anatomical site for 362, and MSI status for 115, including 11 MSI-H tumors. These associations place the receptor-side programs within the bulk multicompartment ecology of sporadic CRC; because CMS classification and measured tumor purity were unavailable, they do not establish CAC specificity or tumor-cell-intrinsic effects.

The published GeoMx DSP CAC study [15] provided complementary spatial context from 16 IBD colectomy patients, including 108 nonsegmented and 26 segmented regions after quality control (Figure 6E). Its collagen-rich, epithelial, immune, and MYC-associated regional features illustrate CAC tissue heterogeneity, but unavailable ROI-level matrices precluded independent module rescoring. Finally, GSE158826 provided an exploratory AOM-DSS perturbation stress test across 10 groups with 3 independent mice per group: 0 of 26 module tests passed FDR correction in the two principal genotype-specific spermidine contrasts, and pooled correction gave the same result (Supplementary Table S7). The spatial literature and mouse experiment therefore supply context and bounded experimental prioritization, not CAC validation, a stable treatment effect, or a causal chain. Across all layers, Table 3 distinguishes the participant-level association and localization findings from the composition qualifier, negative stress tests, and contextual analyses.

## 3. Discussion

The first component of this framework is the disturbed luminal ecosystem itself. In HMP2, IBD was accompanied by broad taxonomic and pathway variation, consistent with studies showing that IBD-associated communities differ in both composition and metabolic activity [16] and that host factors, inflammation, and microbial ecology jointly shape fecal metabolite profiles [17]. UC multi-omics has also linked bacterial functional activity to disease severity [18], while pathway-level syntheses emphasize that IBD dysbiosis involves functional genes and microbial pathways rather than isolated taxa alone [19]. These upstream data establish the ecological setting in which injured mucosa responds, but they do not identify the responding cell types or demonstrate synchronized exposure of the mucosa.

Measured fecal metabolomics provided an independent view of the disturbed luminal environment. Variation in inflammatory lipid-like, polyamine-like, SCFA/fermentation-like, bile acid/steroid-like, and tryptophan/indole-related categories is compatible with the established metabolic complexity of IBD [20,21,22]. However, the strict same-participant ≤2-week analysis included only 18 participants and yielded no FDR-supported category-to-host-module association among 66 tests. Stool metabolites integrate diet, microbial activity, transit, medication, inflammation, and absorption, whereas mucosal receptor engagement depends on local concentration, epithelial access, anatomical site, and recipient-cell state. The fecal metabolomic data therefore provide cohort-level metabolic context, while direct mapping from individual metabolites to mucosal receptor-side programs remains to be tested.

Several metabolite-related observations require context-dependent interpretation. Fecal SCFA-related signals do not directly measure epithelial uptake, local butyrate availability, receptor activation, or barrier benefit. Likewise, a broad polyamine-related stool category and an experimental spermidine intervention differ in compound identity, dose, timing, anatomical compartment, and disease stage [23]. A decreased transcriptomic bile-acid response may reflect altered receptor expression, epithelial damage, or inflammatory feedback rather than absence of exposure. These considerations support a recipient-state model of metabolite biology without establishing direct metabolite-to-host mediation.

The strongest host-side evidence was the coordinated participant-level associations among metabolite-sensing, barrier, inflammatory, reactive/glial-adjacent, and receptor-side programs. Thirteen of 20 prespecified participant-mean associations passed FDR correction; 13 of 20 remained supported after diagnosis/inflammation adjustment, and 12 were shared. Particularly prominent relationships connected the SCFA receptor/barrier-response program with the reactive/glial-adjacent and CGRP/adrenomedullin-family contexts, and connected the AHR-response program with the same receptor-side context. These associations are consistent with previous studies describing neuroimmune interactions among epithelial, immune, glial, neuronal, stromal, and vascular compartments during intestinal injury and repair [24,25,26,27]. The bulk-transcriptomic signal does not indicate neuronal expansion or synaptic rewiring; it identifies a receptor-side and downstream host-response state embedded in the injured mucosa.

Experimental and review literature indicates that enteric glia can regulate intestinal immunity, tissue repair, and epithelial barrier function [28,29]. The present data are compatible with that biology but do not establish enteric-glial abundance, expansion, or activation. CXCL10, NOS2, and S100A10 are non-cell-specific inflammatory or injury-responsive genes (Supplementary Figure S5); the inflammation-associated reactive/glial-adjacent label therefore describes a multicellular response context. Spatial and protein-level colocalization with canonical glial and neuronal markers, together with epithelial, myeloid, stromal, vascular, and receptor-side targets, will be required to identify the contributing compartments. HPA annotations support antibody and tissue-assay prioritization for this work, but they do not constitute disease-specific protein validation.

Single-cell localization strengthens the recipient-compartment interpretation. In GSE132465, CGRP/adrenomedullin-family and AHR-related programs were prominent in stromal summaries, the LPS/TLR-associated inflammatory expression score in myeloid summaries, and the cholinergic receptor-side context in epithelial summaries. In GSE214695, CALCRL, RAMP2, and RAMP3 localized mainly to endothelial cells, VIPR1 to epithelial cells, and VIPR2 to stromal cells; these assignments were stable across detection thresholds. None of the 105 participant-aware IBD-versus-healthy comparisons passed FDR correction, indicating that compartment preference was more reproducible than disease-associated remodeling in this cohort. The findings therefore define receptor-bearing compartments for future spatial and functional testing rather than demonstrating ligand–receptor communication or receptor activation.

The UC and colorectal-neoplasia analyses provide disease context for this mucosal architecture. In GSE47908, several programs distinguished controls from established colitis, but no module differed between pancolitis and left-sided colitis after FDR correction, and only three dysplasia samples were available in the processed matrix. GSE236055 likewise yielded no FDR-supported patient-level progressor association. Thus, GSE236055 did not provide independent confirmation of the main findings in the context of UC progression. In 380 unique primary sporadic TCGA COAD/READ tumors, receptor-side scores covaried with stromal, immune, and vascular programs, while the published GeoMx CAC study illustrated spatially heterogeneous epithelial, stromal, immune, and MYC-associated regions [15]. These contextual observations are consistent with broader genomic, microbiome, and metabolome literature on IBD-associated and colorectal carcinogenesis [30,31,32]. Local physicochemical conditions may also modify protein-state behavior; calcium-dependent changes in S100A9 fibril assembly provide one example from a non-intestinal experimental system [33]. This observation is included only as general context and does not establish ionic regulation of the receptor-side programs examined here in IBD or CAC. Together, these analyses support chronic-inflammation and tumor-tissue context; they do not establish a continuous transition from IBD to dysplasia or CAC-specific retention of the receptor-side relationships.

The translational value of this work lies in a prioritized experimental map rather than an immediately deployable diagnostic test. The strongest candidate relationships are the SCFA receptor/barrier program with the reactive/glial-adjacent and CGRP/adrenomedullin-family contexts, together with the AHR–CGRP/adrenomedullin-family relationship. Disease- and site-matched spatial transcriptomics and multiplex immunohistochemistry/immunofluorescence should first determine whether the nominated receptor proteins colocalize with endothelial, epithelial, stromal, neural, glial, and immune markers. Prospectively matched stool and mucosal samples from the same participant and anatomical site should then test local exposure–response relationships, followed by receptor stimulation or inhibition to establish functional dependence. Prospective neoplasia cohorts would be informative only after the spatial and functional components have been reproduced.

Several limitations define the current scope of the study. The contributing datasets contain no matched cross-dataset participants and differ in disease setting, specimen type, platform, sampling design, and independent unit. Although participant-level aggregation reduced repeated-biopsy pseudoreplication in HMP2, the strict host–metabolome analysis included only 18 participants. GSE214695 contained 18 sample/participant units, the processed GSE47908 matrix retained 48 of 60 series samples and only 3 of 6 dysplasia samples, and the AOM-DSS groups contained 3 mice each. TCGA lacked CMS classification and measured tumor purity, and ROI-level expression data were unavailable for independent GeoMx rescoring. The metabolite categories and several host modules remain exploratory expression summaries, and the composition proxies cannot fully distinguish cell abundance from within-cell regulation. Public HPA annotations support protein and assay feasibility, but no new author-generated tissue, protein, or functional experiment was performed. These limitations constrain mechanistic and clinical interpretation while leaving the participant-level covariance and receptor-compartment localization findings as a defined foundation for targeted validation.

## 4. Methods

### 4.1. Study Design and Analytical Rationale

This study used public multi-omic data to test whether IBD-associated microbial and metabolic dysregulation is accompanied by mucosal host-response programs involving receptor-side neuroimmune remodeling and colorectal-neoplasia tissue context. In this cross-sectional study, remodeling was defined by participant-level organization of host-expression programs and compartmental localization of receptor transcripts, not by longitudinal change, receptor activation, or ligand–receptor communication. HMP2/IBDMDB provided the primary participant-level host analysis and microbial context; ST000923 provided measured fecal-metabolite data; GSE132465 and GSE214695 provided CRC and IBD single-cell localization; GSE47908 and GSE236055 provided complementary UC cross-sectional and progressor analyses; TCGA COAD/READ provided sporadic-CRC bulk-tissue context; the published GeoMx DSP CAC study [15] provided literature-level spatial context; GSE158826 provided exploratory AOM-DSS experimental context; and HPA v25.1 provided protein-assay feasibility information. The datasets were analyzed separately using their appropriate participant, patient, specimen, or experimental unit and were not treated as successive stages of one disease course. Figure 1 summarizes the study design, and Table 1, Table 2 and Table 3 report dataset roles, principal estimates, and the interpretations supported by each analysis.

### 4.2. Public Data Acquisition and Preprocessing

HMP2/IBDMDB host transcriptomes comprised 251 mucosal biopsies from 90 participants and were analyzed as log2 counts-per-million after alignment to biopsy metadata. Gene symbols were converted to uppercase, duplicated symbols were collapsed by mean expression, and nonnumeric values were treated as missing. HMP2 taxonomic and pathway profiles were processed separately and represented microbial composition or functional potential rather than measured metabolites. ST000923 comprised 546 stool samples and 596 measured metabolite features. GSE47908 and GSE158826 were analyzed from processed expression matrices with dataset-specific gene annotation. The available GSE47908 matrix contained 48 of the 60 series biopsies: 15 controls, 14 of 20 left-sided-colitis samples, 16 of 19 pancolitis samples, and 3 of 6 dysplasia samples. TCGA COAD/READ was restricted to 380 unique primary tumors. Dataset denominators and availability are reported in Table 1 and Figure 1, Figure 2, Figure 3, Figure 4, Figure 5 and Figure 6.

For the HMP2 metagenomic diagnosis context, each of the 1638 MGX profiles was analyzed as a sample-level observation; repeated samples from the same participant were not collapsed, so this analysis was interpreted descriptively rather than as participant-level inference. After filtering, 178 genus/species taxa and 353 unstratified pathways were tested in the UC-versus-non-IBD, CD-versus-non-IBD, and UC-versus-CD contrasts using two-sided Mann–Whitney U tests. Benjamini–Hochberg correction was applied separately within each diagnosis contrast and feature class, producing 534 taxon and 1059 pathway tests overall. Dysbiosis associations used sample-level Spearman correlations with correction within each clinical-variable family.

### 4.3. Module Definition, Gene Coverage, and Scoring

Modules were defined before the principal host-association tests to represent SCFA receptor/barrier response, tryptophan–indole/AHR response, bile-acid/TGR5–FXR response, LPS/TLR inflammatory response, epithelial injury and repair, inflammation-associated reactive/glial-adjacent response, CGRP/adrenomedullin-family receptor-side context, VIP/cholinergic receptor-side context, tachykinin receptor-side context, myeloid inflammation, CAF/TGF-β/ECM remodeling, endothelial angiogenesis, and immune-organized ecotypes. The compact CXCL10/NOS2/S100A10 score was treated as a non-cell-specific reactive/glial-adjacent context and not as evidence of enteric-glial identity, abundance, expansion, or activation. Exact gene lists and coverage are provided in the Supplementary Materials; modules with incompletely documented provenance or expected direction were treated as exploratory expression summaries.

For each bulk-transcriptomic dataset, available module genes were standardized within that dataset and averaged without imputing absent genes. Scores therefore represented relative within-dataset expression and were not compared as absolute values across platforms. Per-dataset gene coverage was recorded. For cross-validated HMP2 analyses, gene means, standard deviations, zero-variance filtering, imputation, and score construction were estimated in each training fold and then applied to held-out biopsies. Unless otherwise stated, Δ denotes the difference in mean standardized score.

### 4.4. HMP2 Host-Response and Microbial-Context Analysis

HMP2 mucosal transcriptomes served as the primary host layer. Biopsy scores were first summarized by averaging repeated biopsies within each of the 90 participants. The primary estimands were Spearman correlations for 20 prespecified metabolite-sensing–by–receptor-side/reactive host-program pairs at the participant level. The same 20 associations were re-estimated after adjustment for diagnosis and inflammation status, with Benjamini–Hochberg correction applied separately to the primary and adjusted families. Percentile 95% confidence intervals for the participant-mean Spearman correlations were calculated from 1000 bootstrap resamples of the 90 participants, with replacement. Biopsy-level diagnosis and inflammation comparisons were retained as tissue-state context rather than as the primary independent-unit analysis (Figure 3; Table 2).

For microbial context, 246 of 251 host records were linked to an available same-participant MGX profile. Because individual MGX profiles were frequently reused for multiple biopsies, these links were treated as ecological timing matches rather than independent synchronized exposures. Matching sensitivity covered eight timing windows and three analytical strategies, with 70 tests in each of 24 scenarios and 1680 tests overall. Multiplicity was controlled within each scenario, and this layer was used only to assess the boundary of synchronized microbial–host association.

Five-fold participant-grouped ridge regression evaluated whether five receptor-side or neuroimmune expression scores added explanatory information beyond four direct host-expression context scores for barrier injury/repair, myeloid inflammation, CAF/ECM remodeling, and endothelial angiogenesis. All models additionally included diagnosis, inflammation status, biopsy location, and recruitment site. A composition sensitivity added epithelial, myeloid, stromal, and endothelial expression-derived proxy scores to both model sets; these proxies were indicators of tissue composition, not measured cell fractions. Participants were confined to one fold, biopsies from participant i received fitting weight 1/ni, and held-out R^2^ was calculated with equal participant weighting. Uncertainty in the unconditioned ΔR^2^ was assessed with 1000 participant-level bootstrap repetitions, and statistical support with 2000 conditional permutations followed by correction across the four outcomes. The models estimated incremental prediction among host-expression programs and were not clinical prediction or microbial-mechanism models.

### 4.5. Measured Fecal Metabolomics and Temporally Matched Host Analysis

Six exploratory metabolite categories were constructed post hoc from ST000923 by case-insensitive text matching of reported metabolite names. Ninety category memberships covered 87 unique features, with three features assigned to more than one category. Feature values were log2(x + 1) transformed, standardized individually, and averaged without weighting within each category. Diagnosis contrasts used Mann–Whitney U tests, and clinical associations used Spearman correlation. Because the categories were name-based rather than ontology-defined, feature overlap and lexical ambiguity were retained and documented (Figure 2).

Temporally restricted host–metabolome matching required stool and mucosal samples from the same participant within 2 weeks, yielding 18 observations from 18 participants. The primary rule retained the first eligible biopsy in source-row order when biopsies were equidistant; a complementary analysis first calculated participant-week median host scores. Associations between six metabolite categories and 11 host modules generated one 66-test family corrected by the Benjamini–Hochberg method. Category medians, rank-percentile aggregation, curated exclusions, 90 leave-one-feature-out analyses, and feature-level tests were used as sensitivities. These analyses estimated synchronized same-participant covariation, not mucosal exposure, receptor engagement, or mediation.

### 4.6. Single-Cell Receptor-Side Localization

GSE132465 was used to summarize receptor-side and downstream-response programs across major CRC cell classes and tumor-versus-normal compartments. This processed-summary analysis was a localization layer and did not support endothelial localization, physical communication, or causal inference. GSE214695 included 46,702 annotated cells from 18 sample/participant units—6 healthy controls, 6 participants with UC, and 6 with colonic CD. Receptor detection and module localization were summarized at raw UMI thresholds of at least 1, 2, and 3. Disease-group comparisons were calculated within each sample and compartment so that each sample/participant unit contributed once; the 105 comparisons formed the participant-aware disease-comparison family. Cell-level profiles identified receptor-bearing compartments, whereas sample-level tests assessed disease-associated differences. Transcript detection was interpreted as localization, not receptor activity or ligand–receptor contact (Figure 4).

### 4.7. UC Extent, UC Progressor, CRC/CAC Context, and Mouse Analyses

GSE47908 analyses were restricted to the 48 biopsies represented in the processed matrix, including three dysplasia samples. Five 15-module families were corrected separately: the four-group ordinal analysis; the primary dysplasia-excluded control/left-sided-colitis/pancolitis ordinal analysis; pooled colitis versus control; pancolitis versus left-sided colitis; and dysplasia versus pancolitis. Ordinal coding summarized cross-sectional group ordering and was not treated as within-person progression. GSE236055 contained 143 non-neoplastic biopsies resolving to 43 deposited patient IDs—13 progressors and 30 nonprogressors. Because the series description reports 14 progressors but a fourteenth deposited ID could not be resolved, the analysis used the 43 identifiable IDs. Repeated biopsies were reduced to one median score per patient; the 20 prespecified modules and 6 derived axes were tested as separate multiplicity families. These UC datasets addressed inflammatory-state context and progressor-group discrimination, not longitudinal progression or clinical prediction (Figure 5).

TCGA COAD/READ was used to assess whether the identified programs covaried with stromal, immune, and vascular expression states in 380 unique primary sporadic CRC tumors. Rank-residual partial Spearman models incorporated available age, sex, stage, and tumor site, with sensitivity models adding MSI status and an expression-derived CAF proxy. Stage was available for 358 tumors, site for 362, and MSI for 115, including 11 MSI-H tumors. Because CMS classification and measured tumor purity were unavailable, estimates were interpreted as bulk sporadic-CRC context rather than CAC-specific or tumor-cell-intrinsic effects. The published GeoMx DSP CAC study—16 patients, 108 nonsegmented regions, and 26 segmented regions—was used only as literature-level spatial context because ROI-level expression matrices were unavailable for independent rescoring (Figure 6).

GSE158826 was analyzed as an exploratory AOM-DSS experimental context. Each experimental group contained three independent mice. Two genotype-specific spermidine contrasts generated 26 core module tests, which were evaluated using exact two-sided Mann–Whitney tests with multiplicity correction; a pooled analysis was treated as a sensitivity analysis. The small groups and multiplicity-adjusted results precluded confirmatory treatment-effect, mechanistic, or cross-species claims.

### 4.8. Orthogonal Public Protein and Assay-Feasibility Assessment

HPA version 25.1 annotations were used to evaluate public protein detectability, antibody availability, and immunohistochemistry feasibility for selected receptor-side and compartment markers. Genes in the reactive/glial-adjacent score were also compared with canonical glial/enteric-nervous-system, epithelial, immune, stromal, and vascular markers. HPA information was used to prioritize future assays and was not treated as disease-state protein evidence, receptor activation, spatial colocalization, or experimental validation.

### 4.9. Statistical Analysis

Unless otherwise specified, between-group comparisons used rank-based tests and continuous associations used Spearman correlation. Benjamini–Hochberg correction was applied separately within each prespecified analysis family, with FDR <0.05 defining statistical support. For repeated-biopsy datasets, the participant or patient ID defined the independent biological unit through aggregation, grouped resampling, or clustered modeling as specified above. Exact two-sided Mann–Whitney tests were used for n = 3-per-group mouse contrasts. Nominal small-sample findings were descriptive and were reported with their direction and denominator. Analyses were implemented in Python 3.11.15 using pandas 3.0.6, NumPy 2.4.6, SciPy 1.17.1, and scikit-learn 1.9.0.

## 5. Conclusions

In conclusion, this study identifies a cross-sectional receptor-side neuroimmune remodeling pattern in IBD, expressed as participant-level associations between metabolite-sensing and receptor-side host programs together with localization of relevant receptor transcripts across mucosal compartments. The microbiome, metabolomic, and transcriptomic datasets were obtained from multiple independent public cohorts. Together, these associations and receptor-transcript localization findings identify candidate receptor–compartment relationships for future spatial and functional investigation. They do not establish a causal metabolite-to-neuron pathway, longitudinal remodeling or neoplastic progression, CAC-specific validation, or a clinical biomarker. The proposed receptor-side neuroimmune remodeling framework represents a conceptual model intended to guide future experimental studies, including disease-matched spatial localization, functional receptor perturbation, and prospectively matched stool–mucosal investigations.

## Figures and Tables

**Table 1:** Datasets, denominators, analytical roles, and interpretation limits.

Analysis	Dataset or Resource	Independent Unit or Denominator	Analytical Role	Main Result	Interpretation
Microbial ecological context	HMP2/IBDMDB metagenomics	1638 MGX samples; sample-level diagnosis contrasts	Taxonomic composition and pathway-potential context	295/534 taxon and 486/1059 pathway diagnosis contrasts passed within-comparison BH-FDR	Ecological context; repeated participant samples were not collapsed and abundances are not measured metabolites
Participant-aware host-expression association	HMP2/IBDMDB mucosal transcriptome	251 biopsies from 90 participants	Correlation among host expression scores	13/20 participant-mean associations passed BH-FDR; 13/20 remained after diagnosis + inflammation adjustment	Same-transcriptome host-state covariance; no exposure, mediation, or causality
Incremental host-expression prediction	HMP2/IBDMDB mucosal transcriptome	90 participants; participant-grouped cross-validation	Incremental prediction among host expression programs	Positive permutation-FDR-supported ΔR^2^ for 4 outcomes; bootstrap CI excluded zero for barrier, CAF and endothelial, but not myeloid	Host-expression prediction only; not a microbial or metabolite mechanism
Nearest MGX ecological timing analysis	HMP2/IBDMDB mucosal transcriptome + MGX	246/251 host records matched; 96.9% matched MGX reuse	Ecological matching sensitivity	0/1680 pairs passed FDR	No synchronized microbial-to-host association established
Matched fecal metabolome–host analysis	ST000923 + HMP2 host transcriptome	Strict ≤ 2 weeks: 18 samples from 18 participants	Same-participant timing-restricted association	2/66 nominal *p* < 0.05; 0/66 BH-FDR < 0.05	No FDR-supported synchronized association; fecal abundance is not mucosal exposure
Exploratory metabolite-category construction	ST000923	596 features × 546 samples; 90 memberships covering 87 features	Post hoc name-regex category summaries	Mean/median/rank/curated and all 90 leave-one-feature-out scenarios remained 0/66 FDR at ≤2 weeks; 0/990 feature tests passed FDR	Exploratory categories; no established biochemical module or synchronized coupling
IBD single-cell localization	GSE214695	46,702 cells; 18 participant-sample units (6 HC, 6 UC, 6 CD)	Cell/compartment localization	0/105 participant-aware disease comparisons passed BH-FDR; receptor transcripts showed compartment localization	Localization only; no ligand–receptor contact, CAC specificity, prediction, or causality
Cross-sectional UC extent context	GSE47908	48 biopsies: 15 control, 14 left-sided, 16 pancolitis, 3 dysplasia	Cross-sectional sensitivity	Dysplasia-excluded ordinal family 11/15 FDR; pancolitis vs. left-sided 0/15; dysplasia vs. pancolitis 0/15	Primarily control-versus-colitis separation; no longitudinal progression or dysplasia confirmation
UC progressor comparison	GSE236055	143 biopsies; 43 deposited IDs: 13 progressors, 30 nonprogressors	Patient-level progressor comparison	0/20 prespecified modules and 0/6 derived axes passed BH-FDR; series text reports 14 progressors	Deposited-ID analysis uses 13 progressors; no biomarker, prospective prediction, or causality
Sporadic CRC contextual compatibility	TCGA COAD/READ	380 unique primary tumors	Bulk-tissue context	Stage 358/380; site 362/380; MSI 115/380 (11 MSI-H); CMS and purity unavailable	Context only; no CAC confirmation, purity/CMS independence, or tumor-cell-intrinsic inference
Mouse experimental context	GSE158826 AOM-DSS ± spermidine/Smox	30 mice; 10 groups; n = 3 independent mice/group	Exploratory experimental context	0/26 core module tests passed FDR; pooled sensitivity also 0	No confirmatory treatment effect, cross-species confirmation, or stable mechanism
Public protein/IHC feasibility	Human Protein Atlas v25.1	Gene-level public annotation	Assay feasibility	Public protein/antibody annotations prioritize future tissue assays	Feasibility only; not disease-state protein evidence in the analyzed cohorts
CRC single-cell localization	GSE132465	Processed CRC single-cell summaries	Receptor-side and downstream-program localization	Stromal, epithelial, myeloid, T-cell, B-cell and mast-cell localization context	Localization only; no endothelial claim from this dataset, contact inference, or causality
Published CAC spatial context	GeoMx DSP CAC study	16 patients; 108 nonsegmented and 26 segmented regions	Literature-level spatial context	Published collagen-rich, epithelial, immune and MYC-associated regional features	ROI matrices unavailable; no independent rescoring or validation

**Table 2:** Participant-level associations and principal quantitative results.

Analysis	Dataset	Comparison or Quantity	Independent Unit	Estimate	Statistical Support	Interpretation
Participant-aware correlation	HMP2	SCFA receptor/barrier response vs. inflammation-associated reactive/glial-adjacent response	90 participants	Spearman ρ = 0.727; 95% bootstrap CI 0.602 to 0.817	BH-FDR = 1.01 × 10^−14^ (20-test family)	Participant-level host-expression covariance
Participant-aware correlation	HMP2	SCFA receptor/barrier response vs. CGRP receptor-side score	90 participants	Spearman ρ = 0.552; 95% bootstrap CI 0.400 to 0.679	BH-FDR = 1.15 × 10^−7^	Participant-level host-expression covariance
Participant-aware correlation	HMP2	AHR-response context vs. CGRP receptor-side score	90 participants	Spearman ρ = 0.531; 95% bootstrap CI 0.364 to 0.681	BH-FDR = 3.18 × 10^−7^	Participant-level host-expression covariance
Participant-aware correlation	HMP2	AHR-response context vs. VIP receptor-side score	90 participants	Spearman ρ = 0.264; 95% bootstrap CI 0.045 to 0.452	BH-FDR = 0.0216	Participant-level host-expression covariance; modest effect
Participant-aware correlation summary	HMP2	20 prespecified host-score pairs	251 biopsies/90 participants	13/20 FDR-supported using participant means; 13/20 after diagnosis + inflammation adjustment; 4 biopsy-level hits were lost	BH within each 20-test family	Participant-aware estimates replace biopsy-level headline correlations
Incremental host-expression prediction	HMP2	Barrier outcome	Participant-grouped pipeline	ΔR^2^ = 0.307; bootstrap CI 0.044 to 1.368	Permutation BH-FDR = 0.000500	Incremental host-expression prediction; CI excludes zero
Incremental host-expression prediction	HMP2	Myeloid outcome	Participant-grouped pipeline	ΔR^2^ = 0.054; bootstrap CI −0.086 to 0.163	Permutation BH-FDR = 0.000500	Permutation result positive, but bootstrap uncertainty includes zero
Incremental host-expression prediction	HMP2	CAF outcome	Participant-grouped pipeline	ΔR^2^ = 0.251; bootstrap CI 0.109 to 0.510	Permutation BH-FDR = 0.000500	Incremental host-expression prediction; CI excludes zero
Incremental host-expression prediction	HMP2	Endothelial outcome	Participant-grouped pipeline	ΔR^2^ = 0.334; bootstrap CI 0.203 to 0.678	Permutation BH-FDR = 0.000500	Incremental host-expression prediction; CI excludes zero
Matched cross-layer analysis	HMP2 MGX	Nearest same-participant MGX–host matching analysis	246/251 host records matched; 1680 tests	0/1680 FDR-supported pairs; 96.9% matched MGX reuse	0 FDR-supported results	Ecological matching does not establish synchronized regulation
Matched cross-layer analysis	ST000923 + HMP2	Strict same-participant ≤2-week metabolite-category–host tests	18 participants; 66 tests	2/66 nominal *p* < 0.05	0/66 BH-FDR < 0.05	No synchronized cross-layer association passed FDR correction
Localization	GSE214695	Participant-aware disease comparisons	46,702 cells/18 participant-sample units	0/105 comparisons passed BH-FDR; compartment localization retained	0/105	Localization without disease-differential or contact inference
Cross-sectional UC extent context	GSE47908	Dysplasia-excluded control/left-sided/pancolitis ordinal family	45 biopsies; 15 modules	11/15 FDR-supported; 10 positive, 1 negative	11/15	Cross-sectional UC context
Direct UC group comparison	GSE47908	Pancolitis vs. left-sided; dysplasia vs. pancolitis	16 vs. 14; 3 vs. 16	0/15 and 0/15 FDR-supported	0/15 in each family	Cross-sectional UC context
UC progressor comparison	GSE236055	Patient-primary prespecified modules and derived scores	43 deposited IDs: 13 progressors/30 nonprogressors	0/20 prespecified modules; 0/6 derived axes	0 discoveries in both families	Series text says 14 progressors; analysis uses 13 deposited IDs
CRC tissue context	TCGA COAD/READ	Receptor-side vs. stromal/immune ecology scores	380 primary tumors	35/40 unadjusted; 34/40 core adjusted; 31/40 core + MSI complete case	BH within model	Bulk-tissue context; CMS/purity unavailable
Experimental context	GSE158826	Two genotype-specific AOM-DSS spermidine contrasts	n = 3 independent mice/group; 26 core tests	0/26 FDR-supported; pooled correction also 0	0 FDR-supported results	Exploratory directions only
Robustness analysis	ST000923	Mean/median/rank/curated categories, 90 leave-one-feature-out scenarios and 990 feature tests	Strict ≤ 2 weeks: 18 participants	All aggregation and LOO scenarios: 0/66 FDR; feature tests: 0/990 FDR	0 FDR-supported results	Name-regex categories remain exploratory
Composition sensitivity	HMP2	Barrier outcome	Participant-grouped pipeline	Unconditioned ΔR^2^ = 0.307; composition-conditioned ΔR^2^ = 0.034	Sensitivity analysis	Attenuation places incremental signal within multicompartment tissue ecology
Composition sensitivity	HMP2	CAF/ECM outcome	Participant-grouped pipeline	Unconditioned ΔR^2^ = 0.251; composition-conditioned ΔR^2^ = −0.001	Sensitivity analysis	Attenuation places incremental signal within multicompartment tissue ecology
Composition sensitivity	HMP2	Endothelial outcome	Participant-grouped pipeline	Unconditioned ΔR^2^ = 0.334; composition-conditioned ΔR^2^ = 0.005	Sensitivity analysis	Attenuation places incremental signal within multicompartment tissue ecology
Composition sensitivity	HMP2	Myeloid outcome	Participant-grouped pipeline	Unconditioned ΔR^2^ = 0.054; composition-conditioned ΔR^2^ = −0.010	Sensitivity analysis	Attenuation places incremental signal within multicompartment tissue ecology

**Table 3:** Interpretive scope of the integrated analyses.

Analysis Type	Data Source	Quantity Evaluated	What the Result Supports	What Remains Untested	Use in This Study
Correlation	HMP2 host transcriptome	Participant-mean and adjusted covariance among host expression scores	Participant-aware organization of host response states	Exposure, cellular communication, mediation and causality	Primary association with caution
Incremental host-expression prediction	HMP2 host transcriptome	Leakage-controlled incremental ΔR^2^ under participant-grouped resampling	Additional prediction among host expression programs; strongest for barrier, CAF and endothelial outcomes	Microbial/metabolite mechanism, transportability and clinical utility	Prediction within the host-expression layer
Matched microbial/metabolite–host association	HMP2 MGX; ST000923 + HMP2	Nearest MGX timing/reuse and strict ≤2-week measured-metabolite-category associations	No FDR-supported synchronized association in the tested matching analyses	Direct regulation, mucosal exposure, receptor engagement and absence of a biological relation	Matched cross-layer result
Localization	GSE214695	Cell/compartment detection and participant-aware disease comparisons	Recipient-side compartment localization	Ligand–receptor contact, neural expansion, CAC specificity and prediction	Localization only
Cross-sectional contextual compatibility	GSE47908	Control/colitis extent ordinal and direct group contrasts	Inflammation/extent-associated cross-sectional tissue context	Longitudinal progression, graded left-sided-to-pancolitis change and dysplasia-specific effect	Context with small dysplasia subgroup
UC progressor comparison	GSE236055	Patient-primary progressor vs. nonprogressor score differences	No FDR-supported patient-level progressor-group difference	Biomarker performance, prospective prediction, clinical utility and causality	No participant-level confirmation
CRC tissue context	TCGA COAD/READ	Bulk receptor-side/ecology co-variation with available clinical adjustments	Compatibility with sporadic CRC stromal/immune tissue context	CAC specificity, CMS/purity independence and tumor-cell-intrinsic effect	Contextual interpretation
Experimental context	GSE158826 AOM-DSS	Genotype-specific spermidine contrasts in n = 3 mice/group	Hypothesis-generating cross-species experimental context	Stable treatment effect, mechanism and human relevance	Exploratory only
Assay feasibility	Human Protein Atlas	Public protein and antibody/IHC annotation availability	Prioritization of future tissue-level assays	Disease-state protein abundance, spatial colocalization and cohort-specific experimental confirmation	Feasibility only
Integrated interpretation	All layers	Complementary analyses in datasets without matched cross-dataset participants	Convergent association, localization, and disease-context evidence	A continuous microbiota→metabolite→neuroimmune→dysplasia/cancer mechanism	Not tested in the current data
CRC single-cell localization	GSE132465	Processed compartment-level receptor/response summaries	CRC mucosal compartment context	Endothelial localization, ligand–receptor contact and causality	Localization context
Published spatial context	GeoMx DSP CAC study	Published region-level spatial features	Rationale for disease-matched CAC spatial analysis	Independent module rescoring and CAC mechanism validation	Literature-level context

## Data Availability

The datasets analyzed in this study are publicly available through HMP2/IBDMDB, Metabolomics Workbench (ST000923), GEO (GSE132465, GSE214695, GSE47908, GSE236055, and GSE158826), TCGA COAD/READ, and the Human Protein Atlas. The corresponding analytical results are provided in the article and Supplementary Tables S1–S12. The analysis scripts used in this study are available from the corresponding author upon reasonable request.

## Supplementary Materials

The following supporting information can be downloaded at: https://www.mdpi.com/article/10.3390/ijms27209179/s1.
